# Balance Expertise Is Associated with Superior Spatial Perspective-Taking Skills

**DOI:** 10.3390/brainsci11111401

**Published:** 2021-10-24

**Authors:** Kirsten Hötting, Ann-Kathrin Rogge, Laura A. Kuhne, Brigitte Röder

**Affiliations:** Biological Psychology and Neuropsychology, Universität Hamburg, Von-Melle-Park 11, 20146 Hamburg, Germany; ann-kathrin.rogge@uni-hamburg.de (A.-K.R.); laura.andrea.kuhne@uni-hamburg.de (L.A.K.); brigitte.roeder@uni-hamburg.de (B.R.)

**Keywords:** motor expertise, balance, spatial cognition, physical activity

## Abstract

Balance training interventions over several months have been shown to improve spatial cognitive functions and to induce structural plasticity in brain regions associated with visual-vestibular self-motion processing. In the present cross-sectional study, we tested whether long-term balance practice is associated with better spatial cognition. To this end, spatial perspective-taking abilities were compared between balance experts (*n* = 40) practicing sports such as gymnastics, acrobatics or slacklining for at least four hours a week for the last two years, endurance athletes (*n* = 38) and sedentary healthy individuals (*n* = 58). The balance group showed better performance in a dynamic balance task compared to both the endurance group and the sedentary group. Furthermore, the balance group outperformed the sedentary group in a spatial perspective-taking task. A regression analysis across all participants revealed a positive association between individual balance performance and spatial perspective-taking abilities. Groups did not differ in executive functions, and individual balance performance did not correlate with executive functions, suggesting a specific association between balance skills and spatial cognition. The results are in line with theories of embodied cognition, assuming that sensorimotor experience shapes cognitive functions.

## 1. Introduction

The study of experts who achieved very high levels of perceptual and sensorimotor performance in their field after years of extensive training has a long tradition in psychology and neuroscience, revealing fundamental principles of skill acquisition and their underlying neuronal mechanisms [1,2]. Athletes, in particular, have been extensively studied in this context, as they acquire very specific sensorimotor skills over years of regular and structured training [3,4]. For instance, Land and McLeod [5] showed that professional cricket batsmen were superior in judging when and where the ball will hit the ground compared to amateur players. The batsmen’s superior performance was specifically linked to reduced latencies of their initial saccade. Elite basketball players were reported to better anticipate other basketball players’ shots, but not soccer kicks, compared to non-experts, and the enhanced anticipatory skills correlated with enhanced excitability of their motor cortices [6].

It is a matter of debate whether enhanced sports-specific skills transfer to the more general cognitive functions assessed with psychometric tests. Visuo-spatial tasks, and mental rotation in particular, are the most studied tasks in this context. Several studies showed enhanced mental rotation skills in experts in combat sports, gymnastics and dancing compared to athletes of other disciplines or non-athletes, with the largest effect sizes for combat sports (for a meta-analysis, see [7]). The findings of expert studies in sports have been discussed in the context of embodied cognition. According to the embodied cognition framework, sensorimotor interactions with the environment play an important role in the development and maintenance of higher cognitive skills [8]. Exercise-related cognitive benefits may be due to a stimulation of overlapping brain networks involved in both the sensorimotor practice and specific cognitive processes, which in turn might transfer to improved performance in psychometric tests addressing these cognitive functions, such as mental rotation. Athletes performing combat sports or gymnastics are highly trained in performing and imaging their own body transformations, even in very unfamiliar body positions [9]. In addition, in combat sports, it is crucial to represent an opponent’s body in space relative to one’s own body to anticipate the opponent’s movements. Thus, enhanced mental rotation skills, measured with psychometric tests in martial artists (combat sports) and gymnasts, might indicate a transfer of skills acquired during motor training, which is in line with predictions of embodied cognition theories.

Gymnasts have been shown to outperform athletes of many other disciplines in balance skills [10]. Balancing requires a rapid and continuous integration of vestibular, somatosensory and visual signals. The integration of these signals is not only important for postural control, but seems to be essential for cognitive functions such as self-motion perception, body self-consciousness, spatial navigation and spatial memory [11]. For instance, spatial memory deficits have been reported in patients with peripheral vestibular lesions [12]. Experimental vestibular stimulation has been shown to impair performance in perspective-taking and mental rotation tasks [13,14]. The vestibular system has widespread cortical connections to brain regions known to be involved in spatial cognition, like the posterior parietal cortex, the temporo-parietal junction, the retrosplenial cortex and the hippocampus [11]. Thus, one might hypothesize that physical exercise, such as balancing, which stimulates particularly vestibular pathways, should have an impact on visuo-spatial skills. In line with this assumption, Dordevic et al. [15] reported improvements in a spatial orientation task after one month of balance training on a slackline. Rogge et al. [16] showed that 12 weeks of a balance training compared to relaxation training improved not only dynamic balance performance, but also memory and spatial cognition in healthy adults. These cognitive effects were rather specific for spatial skills, while no group differences were found in executive functions or response speed. Moreover, cortical thickness was increased in the balance group in brain regions associated with visual and vestibular self-motion processing, such as the superior temporal cortex, visual association cortices, the posterior cingulate cortex, the superior frontal sulcus and the precentral gyri [17]. In a cross-sectional study, professional dancers and slackliners were found to have larger grey matter volumes in the posterior hippocampus compared to non-balance experts [18]. On a behavioral level, balance experts outperformed non-experts in a hippocampus-dependent configurational learning task, but not in spatial memory and navigation tasks [18].

Taken together, athletes with a history of extensive training in combat sports, gymnastics and dancing have been found to outperform athletes of many other disciplines and non-athletes in visuo-spatial tasks requiring mental transformation of their own body or objects in space. These enhanced skills might be mediated by practicing mental rotation during training. This hypothesis is further supported by the observation that users of sign language, which provides extensive practice in visuo-spatial processing, typically have superior mental rotation skills [19,20]. In addition, training involving the stimulation of vestibular networks, which are involved in visuo-spatial processes, might contribute to better mental rotation skills in athletes performing sports activities with high balance demands.

Most of the published studies have found higher mental rotation abilities in athletes practicing gymnastics and combat sports compared to sedentary groups, but not when contrasting their performance with athletes of other disciplines [21,22,23]. Only a few studies have shown better mental rotation skills in gymnasts compared to athletes of other disciplines, after controlling for overall physical activity [9,24]. It remains an open question whether the reported superior spatial cognitive skills in gymnasts indicate an association between practicing a specific type of exercise and cognitive functions or are due to an overall high level of physical activity.

During the last two decades, numerous epidemiological and cross-sectional studies have reported better cognitive and academic performance in physically active people compared to sedentary individuals (e.g., [25,26,27,28,29,30]). Furthermore, randomized intervention studies showed beneficial effects of regular exercise training on a wide range of cognitive functions, including memory, executive functions, visuo-spatial skills and attention [31,32,33]. Most published intervention studies implemented aerobic exercise training. Consequently, some authors suggested that aerobic exercise selectively improves executive functions, especially in older populations [34,35]. However, executive functions have been shown to be enhanced by complex motor training, which did not improve cardiovascular fitness [36]. A recent meta-analysis integrated 80 intervention studies on the effects of physical exercise on cognition and found the largest cognitive benefit from exercise programs that improved fine and gross-motor body coordination and balance skills over any other form of physical exercise. Overall, effect sizes did not differ significantly between cognitive domains, suggesting a rather overarching effect of physical exercise on cognition [37]. Taken together, both epidemiological and training studies in previously sedentary healthy adults have established a reliable link between regular physical exercise and better cognitive functioning.

However, it is unknown whether enhanced spatial skills in athletes highly trained in combat sports or gymnastics compared to sedentary participants are predominantly due to a high amount of physical exercise, practicing mental rotation during training by anticipating others’ movements, mental imagery of one’s own body movements, a stimulation of vestibular networks by balance training or a combination of these factors. The goal of the present study was to further unravel the specific contribution of balance skills on spatial cognitive performance. Therefore, we recruited balance experts who were regularly engaged in balance activities on their own, that is, without an opponent or partner. Moreover, in order to control for the balance experts’ typical high overall fitness, we included endurance athletes as an active control group. We hypothesized that balance experts have specific advantages in visuo-spatial skills, both compared to endurance athletes and compared to sedentary individuals. All participants were tested on dynamic balance, spatial perspective-taking and executive functions. We additionally hypothesized that better individual balance skills are associated with better performance in the perspective-taking task. Both this correlation, as well as the overall higher performance of the balance group, were expected to be specific for the perspective-taking skills; that is, they were not expected for executive functions.

## 2. Materials and Methods

### 2.1. Participants

An a priori sample size calculation was performed with G*Power 3.1.9.2 [38]. Based on the meta-analysis of Voyer and Jansen [7], we expected a medium effect size for enhanced visuo-spatial skills in balance experts. Such an effect size can be statistically detected in a one-way Anova (three groups) with a total sample size of 159 participants (power = 0.80, alpha = 0.05). As described in detail in the next paragraphs, the data of some participants had to be discarded for the group analysis because the participants could not be unambiguously categorized as balance experts and endurance athletes, did not follow task instructions in the perspective-taking task or their data were classified as outliers. Thus, the achieved power to detect a medium effect size in the group analysis (*n* = 133) for the main outcome measure (deviation error in the perspective-taking task) was 0.72.

Individuals between 18 and 50 years of age were eligible for the study if they reported practicing either balance activities or endurance sports for at least four hours a week during the last two years. Athletes were recruited in sports clubs, at running events, through word-of-mouth, using announcements in sports-specific social media groups and via a university recruitment platform for psychological studies. Activities in the balance group included acrobatics, ballet dancing, skateboarding, bouldering, unicycling, slacklining, freestyle taekwondo (without opponent), dancing (solo dancing only), gymnastics, trampoline, tricking and yoga. Activities in the endurance group included cycling, running and aerobic fitness training. Individuals practicing team sports, ball games, combat sports with an opponent or dancing with a partner were not eligible for this study. Furthermore, participants were not considered for the study if they reported practicing both balance activities and endurance activities regularly. No current or past engagement in competitions was required for taking part in the study. Participants were screened in a telephone interview, and 97 participants were invited for testing. During the assessment session, all participants filled in a questionnaire about their physical activities, including their sports activities during the last week (Freiburg Questionnaire of Physical Activity, FQPA, [39]). Moreover, the interviewer assessed their sports activities in detail. Based on data of the FQPA and the second interview, participants were classified as balance experts or endurance athletes. Three of the invited participants did not fulfil the criteria of practicing 4 h a week on a regular basis, and 16 participants could not be unambiguously classified as balance experts or endurance athletes, and their data was thus disregarded for the group analysis. The final sample comprised 78 athletes (40 balance experts and 38 endurance experts). By contrast, the data of all invited athletes (*n* = 97) were considered for the regression analyses exploring the association between balance performance and cognitive measures.

Balance experts and endurance athletes were compared to 59 sedentary participants (20–40 years of age, 43 female). Sedentary participants were recruited for a physical exercise intervention study, which will be reported elsewhere. The data recorded at baseline were included for the present study. Sedentary participants reported less than five exercise sessions a month during the last five years.

None of the participants had any history of neurological disease, and they reported no intake of antidepressant or antipsychotic medication. According to self-reporting, all participants had normal or corrected-to-normal vision and normal hearing abilities. Most of the participants (93%) held an A-level certificate or a university degree.

The local ethical board of the Faculty of Psychology and Movement Science at the University of Hamburg approved the study, and all participants gave written informed consent. Participants received course credit or monetary compensation of 16 € for participation.

### 2.2. Assessments

#### 2.2.1. Balance Test

Balance performance was tested with a stability platform (Stability Platform, Modell 16,030 L, Lafayette Instrument Company, Lafayette, IN, USA). Previous studies have demonstrated a sufficient sensitivity of this method for distinguishing balance experts from professional soccer players, swimmers and non-athletes [40,41].

Participants stood barefoot on an unstable platform with a maximal deviation of 15 degrees to each side. They were instructed to place their hands on their hips, direct their gaze to a fixation cross straight ahead (eyes-open condition only) and to keep the platform in a horizontal position for as long as possible during a 30 s trial. After a practice trial, three 30 s trials with eyes open and three 30 s trials with eyes closed were run, separated by 30 s breaks. Whether participants started with eyes open or eyes closed was counterbalanced across participants. A handrail was available to prevent falls and for use during rest. When participants touched the handrail during a trial, the trial was repeated. Testing was stopped after three unsuccessful attempts. This was the case for one participant in the eyesclosed condition. A built-in digital encoder recorded the time per trial the platform was in the horizontal position (± 3° deviation). The mean time spent in a horizontal position across trials was calculated for each participant, separately for eyes open (EO) and eyes closed (EC).

#### 2.2.2. Perspective-Taking Abilities

The Orienting and Perspective Taking Test (OPT, [42]) was used to assesses the ability to image scenes from different viewpoints, a sub-function of spatial cognition. In this paper-pencil test, participants were shown a picture with seven objects. Their task was to imagine standing at one given object, facing a second object and indicating the direction of a third object. A circle was printed under the scene, with an arrow pointing in the direction of the object the participant was facing. Participants were instructed to draw a second arrow indicating their imagined pointing direction. They were not allowed to turn the page or their own body for viewpoint shifts. The time limit to solve 12 items was set to 5 min. Deviation errors were scored by subtracting the participants’ angle estimates from the correct solutions. If a participant did not answer an item, missing values were replaced with 90°, as reported in [43]. The mean deviation across items was calculated for each participant. Smaller values represent better performance.

Data of four participants were not included in the analyses of the OPT task because two participants did not understand the instructions of the task (*n* = 1 endurance group, *n* = 1 sedentary group) and two participants (balance group) had mean deviation errors of more than three standard deviations above the group mean and were therefore excluded as outliers.

#### 2.2.3. Executive Functions

A computer-based modified version of the Eriksen flanker task [44,45] was used to assess executive control and inhibition. Stimulus presentation and recording of responses were performed using Presentation^®^ Software (Version 14.9, Neurobehavioral Systems, Inc., Berkeley, CA, USA). Five arrows were presented in the middle of a computer screen (white color on black background). Participants were asked to indicate the direction of the middle arrow as fast as possible by pressing a left and right button on a custom-made device. Participants responded with the index and middle fingers of the dominant hand. Each stimulus was presented for 1000 ms, and the inter-stimulus interval was set to 1000 ms. The probability of middle errors pointing to the right versus left was the same. In half of the trials, the arrow in the middle indicated the same direction as the flanker arrows (congruent trials), and in the other half of the trials, the arrow in the middle pointed in the opposite direction as the flanker arrows (incongruent trials). In total, 2 blocks of 50 trials each were run, with a short break between blocks. Congruent and incongruent trials were presented in random order within a block.

Trials with reaction times faster than 200 ms and reaction times slower than 3 standard deviations above the individual mean reaction time were discarded from further analyses. Only correct trials were considered for reaction time analyses. On average, less than 1.5% of the trials were error trials. Data of the Flanker task were missing for four participants due to technical problems (*n* = 2 balance group, *n* = 1 endurance group) and non-compliance with task instructions (*n* = 1 balance group).

#### 2.2.4. Physical Activity Questionnaire

The German version of the “Freiburg Questionnaire of Physical Activity” (FQPA, [39]) was used to assess participants’ general physical activity and their sports activities in particular. The questionnaire encompasses questions about basic physical activities (e.g., walking to work, taking the stairs, gardening), leisure-time activities (dancing, light cycling tours) and sports activities. Hours of activity per week were calculated separately for basic physical activities, leisure-time activities and sports activities. Moreover, metabolic equivalents (MET) per week were calculated to estimate the total energy expenditure associated with physical activities, using the compendium provided by Ainsworth et al. [46]. One participant did not fill in the FQPA.

#### 2.2.5. Verbal Intelligence Test

The German “Mehrfachwahl-Wortschatztest” (MWT-B, [47]) was used to estimate participants’ verbal intelligence. The MWT-B comprises 37 rows with four pseudo-words and one legal German word. Participants have to strike out the legal German word with a pencil. The item difficulty continuously increases from the first to the last row. The test score correlates with the general IQ in healthy adults [47].

### 2.3. Data Analysis

Balance performance and perspective-taking skills were compared between balance experts, endurance athletes and sedentary participants by means of an Analysis of Covariance (Ancova), using age and gender as covariates. The significant main effects of the factor group were followed up with simple contrasts, comparing the balance group against the endurance group and the balance group against the sedentary group.

For executive functions, a 2 × 3 Ancova with the within-subject factor Congruency (congruent vs. incongruent) and the between-subject factor Group (balance vs. endurance vs. sedentary) as well as the covariates age and gender was run to test for differences in reaction times in the flanker task.

Linear regression models were run to test for associations between balance performance and cognitive functions. We ran hierarchical models, entering age and gender as predictors in the first step and balance performance with eyes open in the second step, to test whether balance performance explained additional variance. Separate models with mean deviation error in the OPT and the flanker effect (reaction times in incongruent trials minus congruent trials) as dependent variable were run. Furthermore, we ran models using additional covariates, including hours of sports activities, hours of basic and leisure time activities and verbal IQ, to test whether the revealed associations were explained by the amount of physical activity and participants’ IQ.

Data analyses were performed using IBM SPSS Statistics, Version 25. Figures were generated using the package gglot 2 [48] in R (Version 3.6.0, R [49]).

## 3. Results

### 3.1. Description of Participant Groups

Characteristics of participants considered for the group analyses are provided in Table 1. Sedentary participants tended to be on average younger than endurance athletes. There were significantly more female participants in the balance group and the sedentary group than in the endurance group. Thus, age and gender were included as covariates in the consecutive analyses. Sedentary participants had significantly lower scores in the verbal IQ test compared to both balance experts and endurance athletes. Verbal IQ was not available for fifteen participants (*n* = 5 did not fill in the test due to time reasons, *n* = 10 were non-native German speakers). Therefore, we reported additional analyses in the subgroup of participants for whom data on verbal IQ were available, controlling for verbal IQ. The balance experts and endurance athletes reported spending on average 8.83 h/week, CI = (7.90, 9.75) for sports activities, while sedentary participants reported 0.52 h/week, CI = (0.25, 0.79). Time spent training did not differ between balance experts and endurance athletes, *t*(76) = 0.57, *p* = 0.568, *d* = 0.13. As expected, endurance athletes practiced sports activities with higher metabolic demands than balance experts, resulting in a significant group difference in metabolic equivalents (METs) assigned to the activities reported in the FQPA, *t*(76) = −3.41, *p* = 0.001, *d* = −0.77.

### 3.2. Balance Performance

**Figure 1:**
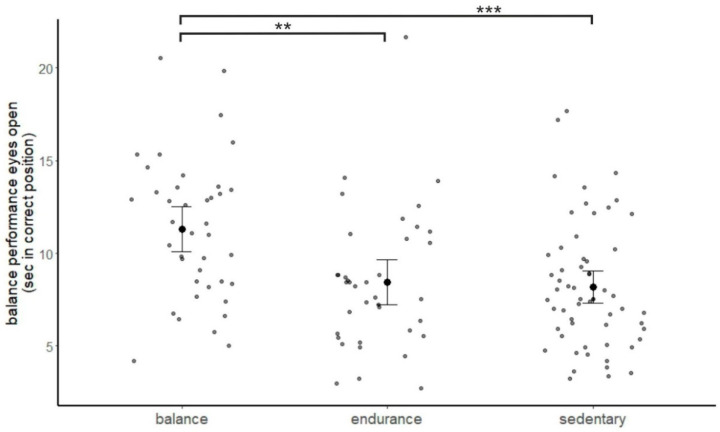
Mean performance on the balance platform in the eyes-open condition, separately for the balance experts, endurance athletes and sedentary participants. Error bars indicate 95% confidence intervals. Data of single participants are plotted in grey. ** *p* < 0.01, *** *p* < 0.001, post hoc contrasts.

Balance performance tested with eyes open was significantly better in balance experts compared to both endurance athletes and sedentary participants, *F*(2, 132) = 13.78, *p* < 0.001, *η*^2^ = 0.173, balance vs. endurance: covariate-adjusted group difference = 2.18, *p* = 0.005, 95% *CI* (0.69, 3.68), balance vs. sedentary: covariate-adjusted group difference = 3.56, *p* < 0.001, 95% *CI* (2.21, 4.90), Figure 1. Groups did not significantly differ in the eyes-closed condition, *F*(2, 131) = 1.43, *p* = 0.244, *η*^2^ = 0.021.

The pattern of results was confirmed when controlling for verbal IQ, *F*(2, 116) = 14.54, *p* < 0.001, *η*^2^ = 0.200, balance vs. endurance: covariate-adjusted group difference = 2.70, *p* = 0.001, 95% *CI* (1.16, 4.24), balance vs. sedentary: covariate-adjusted group difference = 3.88, *p* < 0.001, 95% *CI* (2.44, 5.32).

### 3.3. Perspective-Taking Abilities

**Figure 2:**
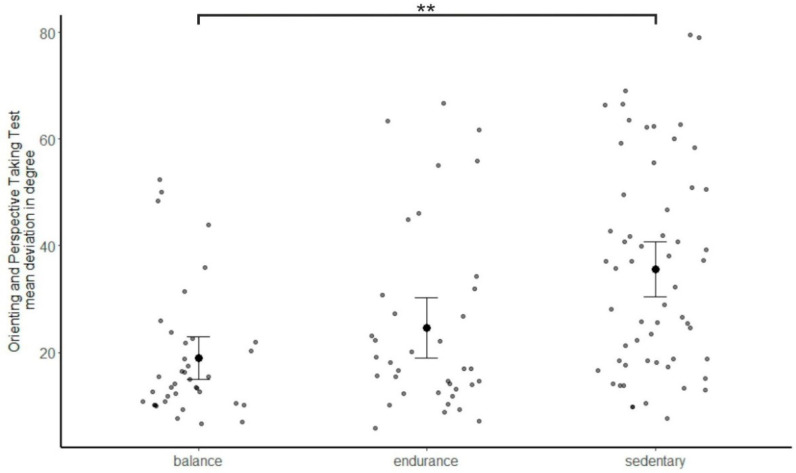
Mean deviation error in the Orienting and Perspective Taking Test, separately for the balance experts, endurance athletes and sedentary participants. Error bars indicate 95% confidence intervals. Data of single participants are plotted in grey. ** *p* < 0.01, post hoc contrast.

Groups significantly differed in the OPT, *F*(2, 128) = 10.56, *p* < 0.001, *η*^2^ = 0.142, with the balance group showing the smallest and the sedentary group the largest mean deviation errors, as shown in Figure 2. Planned contrast confirmed a significantly better performance for the balance group compared to the sedentary group, with a covariate-adjusted group difference = −16.23, *p* < 0.001, 95% *CI* (−23.36, −9.10). The difference between the balance group and the endurance group was not significant, with the covariate-adjusted group difference = −6.16, *p* = 0.130, 95% *CI* [−14.17, 1.84].

The group difference remained significant when controlling for verbal IQ, *F*(2, 113) = 5.92, *p* = 0.004, *η*^2^ = 0.095, balance vs. sedentary group: covariate adjusted group difference = −12.40, *p* = 0.001, 95% *CI* (−19.60, −5.21).

### 3.4. Executive Functions

**Figure 3:**
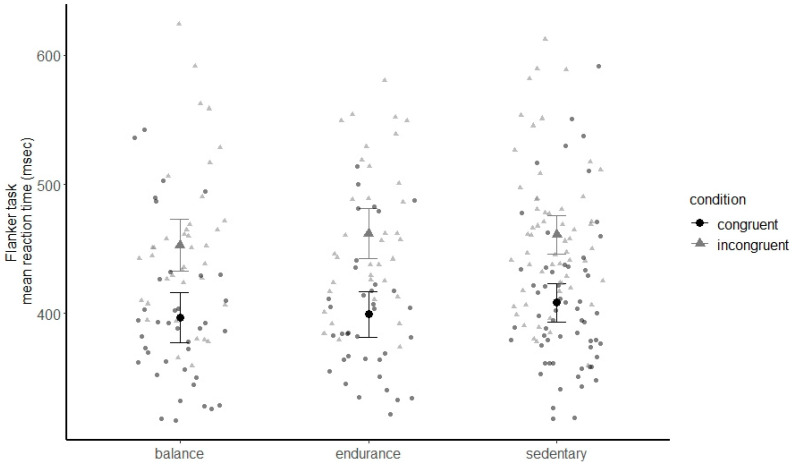
Mean reaction times in the flanker task, separately for congruent versus incongruent trials and for the balance experts, endurance athletes and sedentary participants. Error bars indicate 95% confidence intervals. Data of single participants are plotted in grey.

Participants showed a reliable flanker effect, with faster reaction times in the congruent condition than in the incongruent condition: F(1, 128) = 31.68, *p* < 0.001, *η*^2^ = 0.198; see Figure 3. Neither overall reaction times, Group F(2, 128) = 0.46, *p* = 0.631, *η*^2^ = 0.007, nor the flanker effect differed between groups, Congruency x Group *F*(2, 128) = 1.10, *p* = 0.335, *η*^2^ = 0.017.

Adding verbal IQ as an additional covariate did not change the pattern of results: Congruency *F*(1, 113) = 19.56, *p* < 0.001, *η*^2^ = 0.148, Group *F*(2, 113) = 0.62, *p* = 0.539, *η*^2^ = 0.011, Congruency x Group *F*(2, 113) = 1.01, *p* = 0.367, *η*^2^ = 0.018.

### 3.5. Regression Analyses

**Figure A1:**
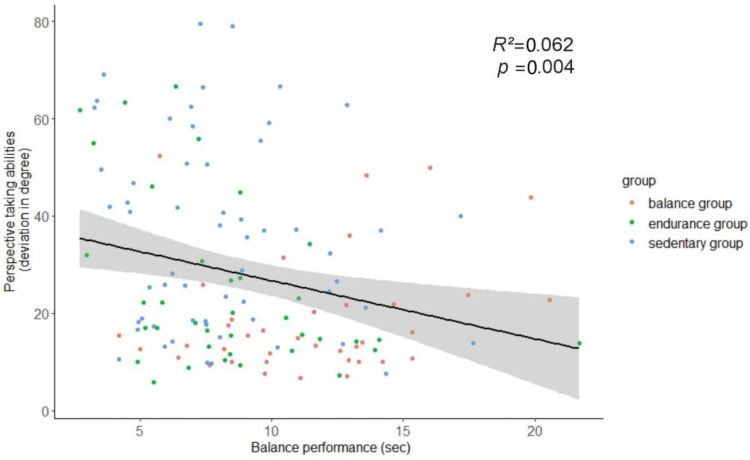
Correlation between the balance performance in the eyes-open condition (seconds in correct position) and perspective-taking skills (OPT task, mean deviation error in degree). N = 133 (*n* = 38 balance group, *n* = 37 endurance group, *n* = 58 sedentary group). Confidence bands indicate 95% confidence intervals.

For regression analyses, data of all assessed participants were considered, including data of those who could not be unambiguously assigned to the balance group, endurance group or sedentary group. By doing so, we tested the overall association between balance expertise and cognitive function in a larger sample and ruled out the possibility that participant exclusion may have biased the reported association between balance expertise and spatial cognition. Additional regression analyses for the subsample considered for the group analyses are provided in Appendix A (Figure A1, Table A1, Table A2).

**Figure 4:**
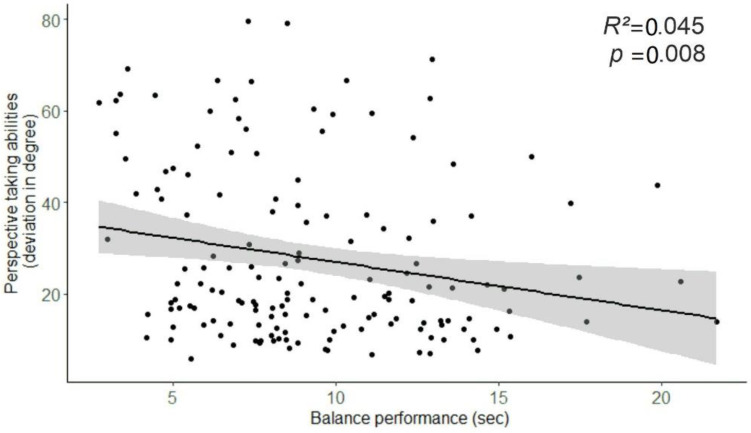
Correlation between balance performance in the eyes-open condition (seconds in correct position) and perspective-taking skills (OPT task, mean deviation error in degree). Confidence bands indicate 95% confidence intervals.

Balance performance was significantly associated with perspective-taking skills (Figure 4), such that the better the performance on the stability platform in the eyes-open condition, the lower the deviation errors in the OPT, standardized *β* (148) = −0.307, *p* < 0.001, adjusted for age and gender. The strength of the association was very similar when adding hours of sports activities/week as an additional covariate, standardized *β* (146) = −0.301, *p* = 0.001; see Table 2.

The flanker effect did not correlate significantly with balance performance, standardized *β* (148) = 0.025, *p* = 0.773, adjusted for age and gender, standardized *β* (146) = −0.027, *p* = 0.760, adjusted for age, gender and hours of sports activities; see Table 3.

We ran further regression models to test whether the reported associations could be explained by participants’ basic and leisure time physical activity and verbal IQ. In one model, age, gender and the sum of basic and leisure time physical activity reported in the FQPA were entered as covariates. In a second model, age, gender and number of correct words in the MWT-B score were entered as covariates. Cognitive measures served as the dependent variable and balance performance with eyes open as the predictor. The additional analyses confirmed a specific association between balance performance and spatial cognition that could not be explained by the amount of physical activity (model 1) and verbal IQ (model 2) (all │*ß*│ > 0.28, all *p* < 0.01). That is, participants with better balance performance had lower deviation errors in the OPT. No associations between balance performance and the flanker effect were found (all │*ß*│ < 0.04, all *p* > 0.7).

## 4. Discussion

In the present cross-sectional study, we provided evidence for a positive association between balance skills and visuo-spatial abilities: Participants who reported practicing balance sports for at least four hours a week during the last two years outperformed sedentary participants in the Object Perspective Taking Test (OPT). There was no significant difference in spatial abilities between endurance athletes and sedentary participants, suggesting that overall physical activity cannot explain group differences in spatial cognition. A regression analysis that included all participants additionally indicated that dynamic balance performance was associated with fewer errors in the perspective-taking task, when controlling for age, gender, verbal IQ and physical activity. The association was specific for the visuo-spatial task, with no group differences in a flanker task assessing response speed and executive functions. Moreover, individual balance performance did not correlate with executive functions.

These results are in line with a meta-analysis on mental rotation skills in motor experts [7], demonstrating overall better performance of motor experts than sedentary participants in mental rotation tasks. However, effect sizes differed considerably for athletes of different disciplines. The largest effect sizes were found for athletes practicing combat sports, followed by those engaging in gymnastics and dancing; effect sizes for endurance athletes did not differ significantly from zero. It has been argued that performing combat sports, gymnastics and dancing requires mental rotation and continuous updating of one’s own and others’ body position in space along all body axes [9,22], while exercise programs of endurance athletes are mostly uniform and predictable. Thus, extensive visuo-spatial processing in combination with motor practice in these disciplines might lead to improved mental rotation abilities, which transfers to better performance in visuo-spatial tasks outside of the sport context. In the present study, we excluded participants practicing combat sports, couple dance and ball sports to narrow down the possible processes that might contribute to better spatial skills in motor experts. Thus, the enhanced perspective-taking skills in the balance experts in the present study are probably less due to extensive training in anticipating the movements of an opponent, partner or object in space, but might be shaped by spatial updating and mental imagery of their own body transformations.

Furthermore, it could be speculated that the vestibular system is a mediating factor. Superior balance skills have been shown for gymnasts and dancers compared to athletes engaging in other physical activities [10,50,51]. Moreover, balance training has been shown not only to improve balance skills but to enhance performance in spatial cognition tests as well [15,16]. In the present study, we investigated the contribution of balance expertise to enhancing spatial skills in athletes by including individuals whose physical activity was associated with high demands on postural control, such as gymnastics, slacklining, acrobatics, ballet, skateboarding, bouldering, unicycling, freestyle taekwondo, dancing, trampolining, tricking and yoga. Moreover, we assessed balance performance explicitly, with a stability platform. Therefore, we demonstrate that individuals engaged in sports activities that place high demands on balance outperformed both endurance athletes and sedentary participants in terms of dynamic balance abilities. Thus, the present data suggest that balance skills should be taken into account when discussing improved visuo-spatial skills in motor experts.

Balancing requires an efficient integration of vestibular, proprioceptive, somatosensory and visual signals. There is growing evidence that the vestibular system essentially contributes to higher-order cognitive functions such as spatial orientation, memory and body self-consciousness [11,52,53]. Visuo-spatial tasks requiring mental spatial transformations are known to activate higher-order visual areas in the occipital lobe, the posterior parietal cortex and the parieto-medial temporal pathway to the hippocampus [54,55]. It should be noted that these regions partially overlap with vestibular cortical networks [11,56,57]. In brain imaging studies, long-term balance expertise and short-term balance training for several weeks have been associated with changes in grey matter volume and cortical thickness in areas receiving vestibular input, such as higher-order visual association areas [17,18], the posterior cingulate cortex [17] and the hippocampus [18,58]. Moreover, balance training has been associated with structural changes in premotor and motor cortices [17,59,60,61], which are known to be activated during mental rotation and mental motor imagery [55,62]. Thus, regular joined stimulation of visuo-vestibular pathways and motor cortices during balance training, which partially overlap with areas important for spatial processing, may contribute to superior performance of balance experts in the spatial task. The results can be interpreted in an embodied cognition framework, which proposes that cognitive processes are based on, or are at least moderated by, sensorimotor processes [63], probably by relying on shared neuronal representations [4]. Enhanced cognitive performance in specific cognitive domains in motor experts compared to novices, such as enhanced perspective-taking skills in balance experts, might thus be the result of long-lasting sensorimotor training [64]. Practicing physical activities with high balance needs is, however, only one factor among those linked to enhanced visuo-spatial skills. For instance, better mental rotation skills compared to controls have been reported for professional orchestral musicians [65] and users of sign language [20].

In the present study, we used a perspective-taking task to measure spatial skills, while previous studies on visuo-spatial skills in motor experts mostly assessed mental rotation abilities. The OPT measures the ability to perform egocentric spatial transformations, i.e., the ability to mentally shift one’s perspective in order to judge the relative position of objects in the environment [42]. In contrast, mental rotation tasks typically measure the ability to imagine movements or the rotation of objects, which requires object-based spatial transformations but not egocentric transformations [42]. Depending on the type of the stimuli and task instructions, the distinction between perspective-taking tasks and mental rotation tasks is less clear-cut. It has been shown that body stimuli in mental rotation tasks trigger perspective-taking strategies. Steggemann et al. [9], for instance, compared experts in artistic gymnastics, aero wheel gymnastics and trampolining to athletes from disciplines that do not involve a lot of spins and turns around the body axes. In this study, the authors used three mental rotation tasks: a letter rotation task with same–mirrored judgments, a body rotation task with same-mirrored judgment and a body rotation task with left–right judgments. The motor experts showed an advantage only in the body rotation task with left–right judgments, which requires an egocentric transformation, i.e., the rotation of one’s own point of view. The authors suggested that the athletes are highly trained in body transformations, even in very unfamiliar body positions, which transfers to the laboratory task of egocentric mental rotation, but not to object-based mental rotation. However, other studies have reported better performance in object-based mental rotation in motor experts [21,23]. Thus, it is still an open question whether there are specific effects of balance expertise on egocentric spatial transformations.

Superior performance of the balance experts in the dynamic balance task compared to endurance athletes and sedentary participants was only found for the eyes-open condition, not for the eyes-closed condition. The balance experts regularly perform their sports with eyes open, making use of visual cues to detect motion displacements and to stabilize posture. Dordevic et al. [58] reported better balance performance in professional ballet dancers compared to age-matched controls in both eyes-open and eyes-closed conditions, but effect sizes were larger for the eyes-open condition. Sedentary participants showed an increase in balance performance after a complex balance training only in the eyes-open testing conditions [66]. Taken together, these results suggest that the effects of motor training on balance skills are more pronounced for the sensory condition that athletes usually experience during their regular training.

We hypothesized that balance training is specially associated with visuo-spatial abilities. Physical exercise is defined as an activity that is “planned, structured, repetitive, and purposeful in the sense that the improvement or maintenance of one or more components of physical fitness is the objective” [67] (pp. 52–53). Physical exercise is a subcategory of physical activity, defined as “any bodily movement produced by skeletal muscles that requires energy expenditure” [67] (pp. 52–53). Both physical exercise and overall physical activity have been linked to enhanced cognitive performance in epidemiological studies [31,32]. In the present study, participants’ amounts of physical exercise training as well as their basic and leisure time physical activity was assessed with a questionnaire. The data showed that the balance group and the endurance group did not differ in the amount of physical exercise nor physical activity in everyday life. Furthermore, the difference between athletes and sedentary participants was much more pronounced for physical exercise (8 h per week) compared to the group difference in basic and leisure time physical activity (2 h per week). Adding basic and leisure time physical activity as additional covariates in the models did not reduce the association between balance skills and visuo-spatial abilities. Therefore, we concluded that there is a specific association between practicing balance sports and visuo-spatial processing that cannot be accounted for by the amount of time spent physically exercising and overall physical activity.

As hypothesized, superior performance of the balance group compared to sedentary participants was specific for the visuo-spatial task and was not observed for executive functions. This is in line with findings of a recent balance training study, which found effects of the balance training compared to a relaxation control training on spatial cognition and memory, but not for executive functions as assessed with the Stroop task [16]. In the present study, the endurance group also did not differ from the sedentary group in executive functions. This was expected on the basis of studies reporting improved executive functions in physically active people compared to sedentary individuals [68,69] and on the basis of training studies proposing beneficial effects of aerobic exercise, particularly on executive functions [34,35,70]. Age has been shown to moderate the influence of exercise on executive functions, with the beneficial effects of aerobic exercise increasing with age in adulthood [71]. It has been suggested that cognitive functions are most sensitive to sensorimotor experiences in phases during which they undergo developmental changes, such as executive functions in childhood and old age [72,73,74,75]. Participants in the present study were between 18 and 50 years of age. In this age range, executive functions are rather stable and at their functional peak [76,77] and thus maybe less likely to be affected by the habitual level of physical exercise.

Studying balance experts allows exploring the effects of regular sensorimotor experience accumulated over years, which is hardly possible to investigate in randomized controlled trials. However, data of the present study are correlational only and do not allow causal interpretations. Reverse causality might account for the reported associations with visuo-spatial abilities influencing whether or not individuals start practicing balance sports and the proficiency level they reach. We showed that balance performance explained variance in visuo-spatial skills that could not be accounted for by participants’ age, gender, verbal IQ and the amount of physical activity. Moreover, participants in the present sample had an overall high level of education, making it unlikely that the reported effects could be explained by group differences in general intelligence. Nevertheless, further variables might have contributed to the association between balance skills and perspective-taking abilities. For instance, playing action video games has been shown to improve spatial cognition [78], which was not assessed in the present study. We measured balance performance with a standardized protocol on a stability platform, but the amount of endurance exercise and participants’ overall physical activity was assessed via self-reporting only, which may be prone to higher measurement errors and biases [79]. Adding standardized assessments of cardiovascular fitness and using wearable activity trackers to draw samples of participants’ physical activity in everyday life might be fruitful approaches to disentangle the contribution of different forms of physical activity to cognitive functions in athletes and sedentary participants in future studies.

The athletes recruited for the present study trained at least four hours per week in their discipline over the past two years. Although some participants reported taking part in sports competitions, this was not an inclusion criterion, and we did not systematically assess how many were engaging in competitions and at which level. Recruiting elite athletes and using standardized procedures for defining expertise level [80] might have increased the power of detecting specific associations between balance training and cognitive functions.

The positive effects of physical exercise on cognition have not only been reported after months or years of training, but after a single bout of cardiovascular exercise [33,81]. One might hypothesize that acute and chronic effects of physical exercise add up when performed regularly and over long periods of time. Studies on acute effects of balance training, however, are lacking. There are some recent results suggesting that the acute cognitive effects of balance exercise do not differ from those after a cardiovascular exercise session [82]. These results should be confirmed in further studies including a control group without training.

Perspective-taking abilities in the present study were assessed using a paper-pencil test, while executive functions were measured with a computer-based flanker task. One might argue that the difference in the assessment modalities might have caused the reported dissociation between cognitive domains. Computerized tests of cognitive function provide very precise measures of reaction times and are therefore more sensitive to individual differences in cognitive performance than paper and pencil tests [83,84]. Thus, if anything, we would have expected more of a correlation between balance expertise and executive functions, which we did not find.

## 5. Conclusions

In the present cross-sectional study, we demonstrate that high balance skills are associated with higher spatial cognitive abilities and that this association cannot be explained by time spent on physical exercise or overall verbal IQ. Moreover, a similar association was not found for executive functions. Practicing balance sports such as gymnastics, acrobatics, dancing and slacklining requires complex body transformations in space and an efficient integration of vestibular, proprioceptive and visual signals. We speculate that the specific skills acquired during balance training transfer to higher spatial cognitive functions in balance experts, probably based on shared neural circuits. From an applied perspective, incorporating balance tasks into physical exercise programs might be a promising approach to increase spatial cognition in athletes and in individuals with deficits in spatial cognition, such as aging populations or patients suffering from neuro-degenerative diseases.

## Figures and Tables

**Table 1:** Participant characteristics (Mean, Sd) for balance experts, endurance athletes and sedentary participants.

	Balance Experts *n* = 40	Endurance Athletes *n* = 38	Sedentary Participants *n* = 59	*p*
Age	27.95 (8.39)	29.71 (7.07)	26.63 (5.33)	0.098 ^3^
Gender (female/male)	25/15	17/21	43/16	0.020 ^4^
Verbal IQ ^1^	29.06 (4.15)	29.11 (3.34)	27.13 (4.23)	0.029 ^3^
Self-reported sports activities ^2^ (hours/week)	9.06 (4.50)	8.58 (3.61)	0.52 (1.04)	<0.001 ^3^
Self-reported sports activities ^2^ (MET)	45.28 (27.84)	67.96 (30.19)	2.48 (4.73)	<0.001 ^3^
Self-reported basic and leisure time activities ^2^ (hours/week)	8.38 (6.96)	8.72 (5.14)	6.44 (4.59)	0.087 ^3^
Self-reported basic and leisure time activities ^2^ (MET)	30.62 (25.11)	34.13 (19.78)	21.91 (15.25)	0.008 ^3^

Note. ^1^ Missing data verbal IQ: *n* = 7 balance group, *n* = 2 endurance group, *n* = 6 sedentary group; ^2^ Missing data FQPA: *n* = 1 balance group; ^3^ Anova; ^4^ Chi-squared test.

**Table 2:** Hierarchical regression model for perspective-taking abilities (OPT task).

Variable	B	95% CI for B		*SE* B	*β*	*R* ^2^	∆*R*^2^
		*LL*	*UL*				
Step 1						0.078	0.078 **
Constant	32.53	20.07	45.00	6.31			
Age	0.057	−0.38	0.50	0.22	0.02		
Gender	−5.56	−11.66	0.55	3.09	−0.15		
Hours sports/week	−0.72	−1.28	−0.16	0.28	−0.21*		
Step 2						0.151	0.073 **
Constant	53.64	36.84	70.44	8.50			
Age	−0.20	−0.64	0.24	0.22	−0.07		
Gender	−9.00	−15.17	−2.81	3.13	−0.24 **		
Hours sports/week	−0.49	−1.04	0.06	0.28	−0.14		
Stability platform eyes open	−1.53	−2.39	−0.68	0.43	−0.30 **		

Notes: CI = confidence interval, *LL* = lower limit, *UL* = upper limit, * *p* < 0.05, ** *p* < 0.01.

**Table 3:** Hierarchical regression model for the flanker effect (RT incongruent–RT congruent).

Variable	B	95% CI for B		*SE* B	*β*	*R* ^2^	∆*R*^2^
		*LL*	*UL*				
Step 1						0.035	0.035
Constant	44.91	28.65	61.18	8.23			
Age	0.22	−0.33	0.78	0.28	0.07		
Gender	5.39	−2.82	13.60	4.15	0.11		
Hours sports/week	0.52	−0.24	1.28	0.39	0.11		
Step 2						0.036	0.001
Constant	47.35	24.68	70.02	11.47			
Age	0.20	−0.39	0.78	0.30	0.06		
Gender	4.97	−3.70	13.64	4.39	0.10		
Hours sports/week	0.55	−0.24	1.34	0.40	0.12		
Stability platform eyes open	−0.18	−1.37	1.00	0.60	−0.03		

Notes: RT = reaction times, CI = confidence interval, *LL* = lower limit, *UL* = upper limit.

## Data Availability

The data presented in this study are available on request from the corresponding author. The data are not publicly available due to privacy concerns.

## Appendix A. Regression Analyses: Subsample Considered for Group Analyses

**Table A1:** Hierarchical regression model for perspective-taking abilities (OPT task).

Variable	B	95% CI for B		*SE* B	*β*	*R* ^2^	∆*R*^2^
		*LL*	*UL*				
Step 1						0.081	0.081 *
Constant	31.14	17.57	44.70	6.85			
Age	0.092	−0.39	0.58	0.25	0.03		
Gender	−3.61	−10.19	2.97	3.33	−0.10		
hours sports/week	−0.88	−1.28	−0.15	0.32	−0.25 **		
Step 2						0.170	0.089 **
Constant	55.18	36.91	73.46	9.24			
Age	−0.24	−0.74	0.26	0.25	−0.08		
Gender	−7.28	−13.86	−0.70	3.33	−0.19 *		
Hours sports/week	−0.59	−1.21	0.02	0.31	−0.17		
Stability platform eyes open	−1.66	−2.55	−0.77	0.45	−0.34 ***		

Note: CI = confidence interval, *LL* = lower limit, *UL* = upper limit, * *p* < 0.05, ** *p <* 0.01, *** *p* < 0.001.

**Table A2:** Hierarchical regression model for the flanker effect (RT incongruent–RT congruent).

Variable	B	95% CI for B		*SE* B	*β*	*R* ^2^	∆*R*^2^
		*LL*	*UL*				
Step 1						0.046	0.046
Constant	47.17	29.89	64.45	8.73			
Age	0.11	−0.49	0.72	0.31	0.03		
Gender	5.81	−2.91	14.54	4.41	0.12		
Hours sports/week	0.68	−0.16	1.53	0.42	0.14		
Step 2						0.047	0.001
Constant	49.67	25.42	73.91	12.25			
Age	0.08	−0.56	0.72	0.32	0.02		
Gender	5.40	−3.81	14.60	4.65	0.11		
Hours sports/week	0.72	−0.16	1.60	0.44	0.15		
Stability platform eyes open	−0.18	−1.41	1.05	0.62	−0.03		

Note: RT = reaction times, CI = confidence interval, *LL* = lower limit, *UL* = upper limit.
